# Simple combination of multiple somatic variant callers to increase accuracy

**DOI:** 10.1038/s41598-023-34925-y

**Published:** 2023-05-25

**Authors:** Alexander J. Trevarton, Jeffrey T. Chang, W. Fraser Symmans

**Affiliations:** 1 School of Biological Sciences, Faculty of Science, University of Auckland, Auckland, New Zealand; 2 Department of Integrative Biology and Pharmacology, The University of Texas Health Sciences Center, Houston, USA; 3 Department of Translational Molecular Pathology, The University of Texas MD Anderson Cancer Center, Houston, USA

**Keywords:** Breast cancer, Cancer genomics

## Abstract

Publications comparing variant caller algorithms present discordant results with contradictory rankings. Caller performances are inconsistent and wide ranging, and dependent upon input data, application, parameter settings, and evaluation metric. With no single variant caller emerging as a superior standard, combinations or ensembles of variant callers have appeared in the literature. In this study, a whole genome somatic reference standard was used to derive principles to guide strategies for combining variant calls. Then, manually annotated variants called from the whole exome sequencing of a tumor were used to corroborate these general principles. Finally, we examined the ability of these principles to reduce noise in targeted sequencing.

## Introduction

Scientists and clinicians who want to identify mutations and other variants in DNA face a wide selection of variant caller algorithms that employ various different approaches. However, discordance and inconsistency exist between bench-marking publications that compare the performances of variants callers. Reported performances may range widely in part due to differences in bench-marking datasets, variant caller parameter settings, and evaluated output metrics. Publications presenting novel variant callers^1–7^ typically show their own caller performing favorably compared to other callers. With no single variant caller emerging as a superior standard, combinations or ensembles of variant callers have appeared in the literature. Ensemble approaches^8–17^ aim to increase confidence in called variants, better differentiate low variant allele frequency (VAF) variants from artefacts and noise, and generate more accurate variant calls for further downstream analysis. Published ensembles differ by the number and types of callers combined, and by method of combination; and across this range, ensemble approaches generally out-perform single variant callers. Despite demonstrated improvements in performance, there has not been wide-spread uptake of ensemble approaches by scientists and clinicians, potentially due to uncertainties in how to choose callers or combine calls. We posited that users may be more willing to improve their variant calling pipelines using simple combinations that are easy to implement and require no further software installations on top of the variant callers. We sought to find generalizable heuristics that are simple enough to spread by word-of-mouth, and thus do not necessitate this paper being read by a wide audience. Goode et al.^18^ reported that a 2/3 majority consensus of the callers MuTect, JointSNVMix2 and SomaticSniper outperformed a 3/3 complete intersection. Brienen et al*.*^19^ described a strategy of using “at least two” of four variant callers. In this paper, we report that majority consensus remains advantageous in combinations larger than three variant callers, when applied to whole genome sequencing (WGS), whole exome sequencing (WES), and targeted amplicon sequencing data. Based on our results, we found that the specific callers used is not critical and recommend a consensus strategy consisting of at least three callers, where variants found by n − 1 callers are accepted. While this study is limited to the datasets here utilized, we expect that these strategies will be tested for generalizability in other datasets over time.

## Results

### Whole genome sequencing

**Figure 1:**
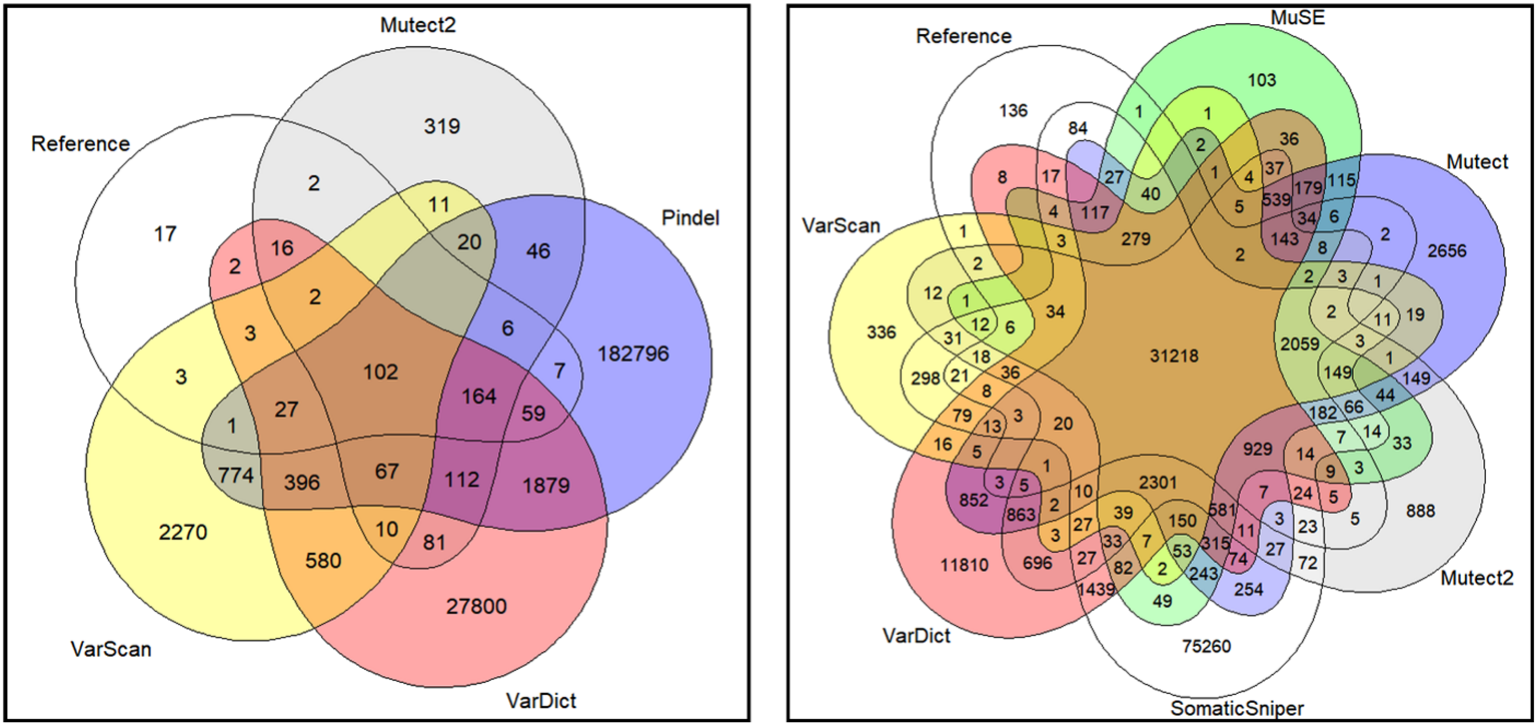
Intersections between reference variants and indels (left) and SNVs (right) called by variant callers. Ellipse and overlapping area sizes are approximately proportional to variant counts.

**Figure 2:**
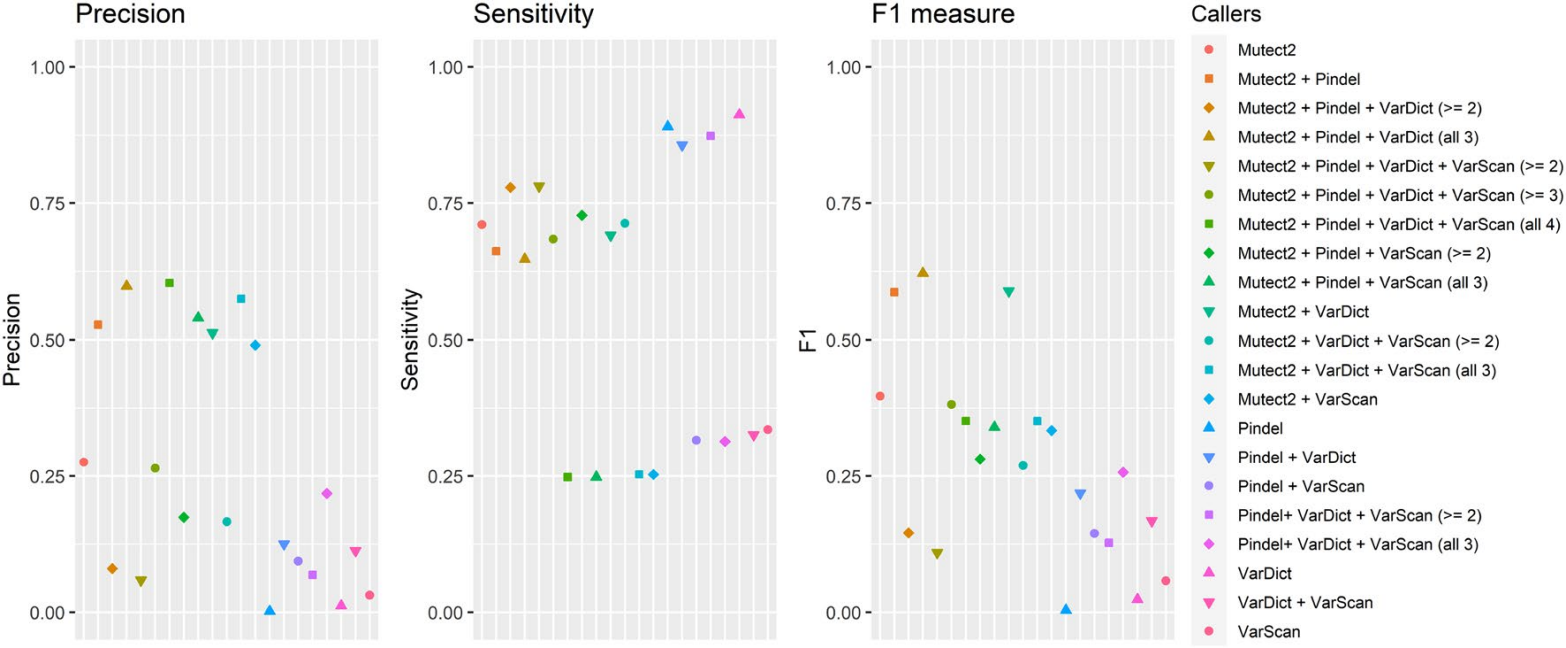
WGS indel caller performance.

**Figure 3:**
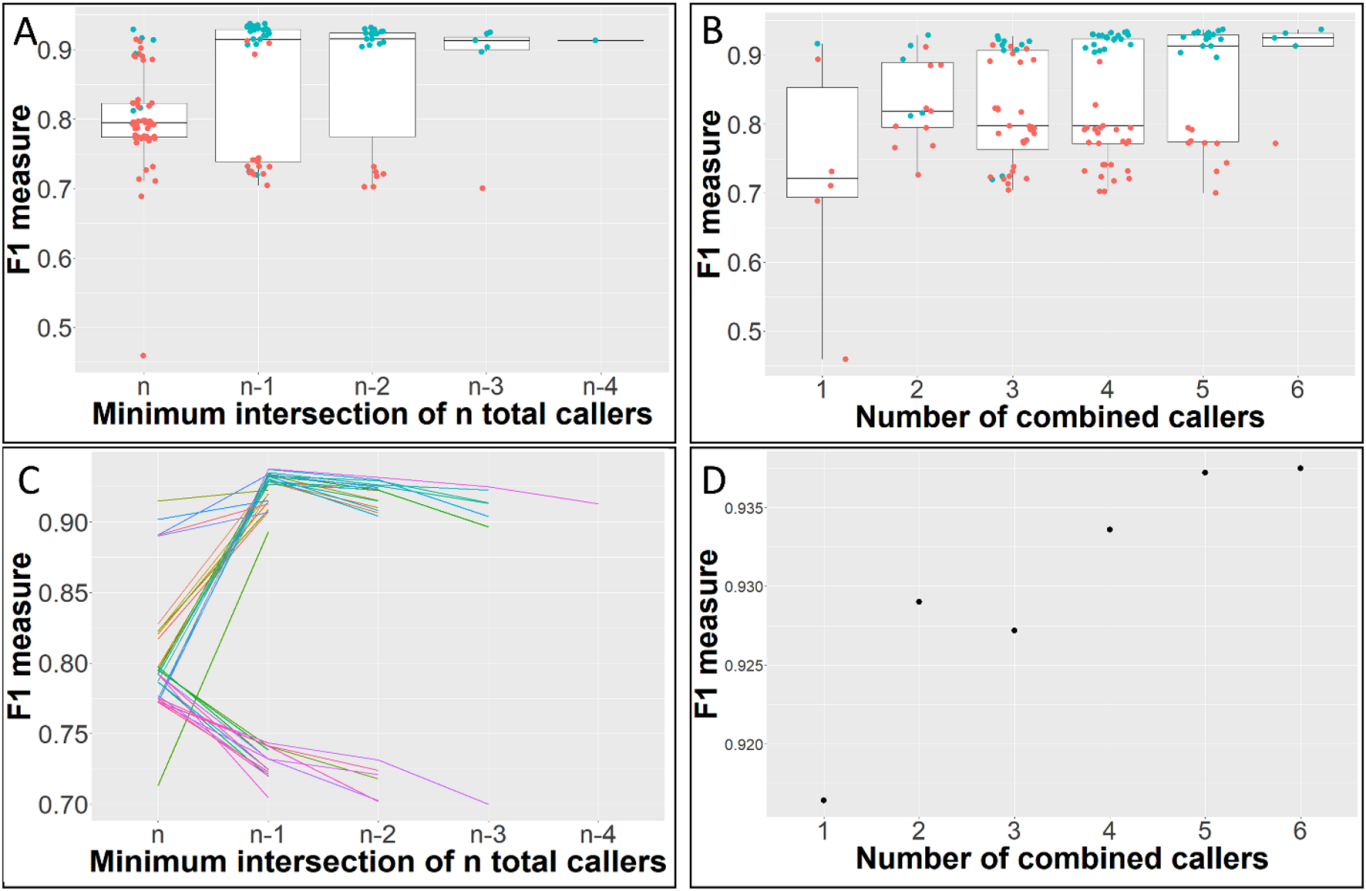
WGS SNV caller performance. (**A**) F1 measures for every possible combination of 1 to 6 SNV callers, grouped by minimum caller intersection thresholds n, n − 1, n − 2, n − 3 and n − 4, where n is the total number of callers combined. The intersection threshold is the minimum number of callers a variant must be called by in order to be accepted. Data points are blue if the combination contains MuSE and, when 3 or more callers are combined, has a minimum caller intersection threshold of n − 1, n − 2, n − 3 or n − 4. (**B**) The same F1 measures, at every minimum intersection threshold, grouped according to the number of total combined callers. Data points are blue if the combination contains MuSE and accepts variants not 3 or more callers are combined) has a minimum caller intersection threshold of n − 1. Data points are blue if the combination contains MuSE and, when 3 or more callers are combined, has a minimum caller intersection threshold of n − 1, n − 2, n − 3 or n − 4. (**C**) F1 measures by minimum intersection thresholds for combinations of 3, 4, 5 and 6 callers, showing the change in F1 as threshold lowers. (**D**) Maximum F1 measures for 1–6 combined callers.

Seven variant callers, with different types of core algorithms (Table 1), were applied to a somatic reference standard^20^. Six of these variant callers were used to call single nucleotide variants (SNVs), and four were used to call indels. 25.4% of indels were called by all four indel callers, with 4.6% of reference indels not called by any caller. 87.8% of SNVs called by all six SNV callers, with only 0.4% of reference SNVs not called by any caller. Intersections between variant callers are visualized approximately in Fig. 1. Figure 2 plots the precision, sensitivity, and F1 accuracy measure (the harmonic mean of precision and sensitivity) of each variant caller and variant caller combination when indels were called. Figure 3 plots the F1 measure of variant caller combinations applied to SNVs, under various intersection thresholds. The intersection threshold is the minimum number of callers a variant must be called by in order to be accepted.

**Table 1:** Variant callers applied to WGS somatic reference standard.

Caller	Variant types	Core algorithm type	Reference
MuSE	SNVs	Markov chain model	^1^
Mutect	SNVs	Allele frequency analysis	^2^
Mutect2	SNVs and indels	Haplotype analysis	^3^
Pindel	Indels	Pattern growth approach	^4^
SomaticSniper	SNVs	Joint genotype analysis	^5^
VarDict	SNVs and indels	Heuristic threshold	^6^
VarScan	SNVs and indels	Heuristic threshold	^7^

Figure 3B,D show median and maximum F1 increasing as the number of combined callers increases. In Fig. 3A, the highest median F1 is obtained by accepting variants called by n − 1 callers. Figure 3C shows that for most caller combinations, the highest F1 measure is obtained at a threshold of n − 1. In Fig. 3A,B, data points are blue if the combination contains MuSE and, when 3 or more callers are combined, has a minimum caller intersection threshold of n − 1, n − 2, n − 3 or n − 4. The observed bimodal distribution in F1 measures can be elucidated by this simple categorization.

### Whole exome sequencing

**Figure 4:**
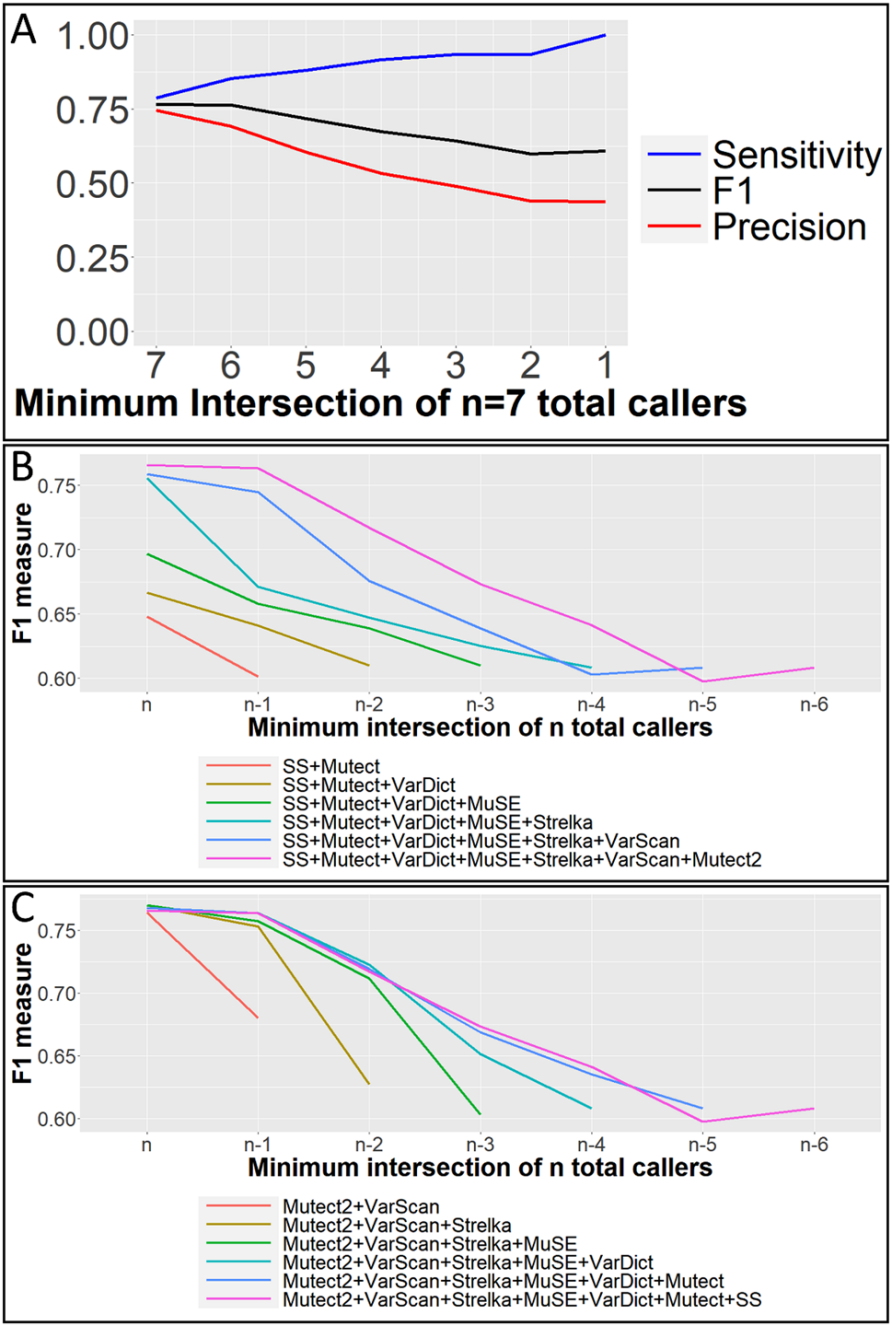
WES SNV caller performance. (**A**) Precision, sensitivity and F1 measures, when variants were accepted as positive when called by a minimum intersection threshold (from 7 to 1) of 7 combined SNV callers. The intersection threshold is the minimum number of callers a variant must be called by to be accepted. (**B**) F1 measures by minimum intersection threshold for caller combinations that increase in size as additional callers are added, ordered from worst to best performing callers. Minimum caller intersection thresholds range from n to n-6, where n is the total number of callers in that combination. *SS* SomaticSniper. (**C**) F1 measures by minimum intersection threshold for caller combinations that increase in size as additional callers are added, ordered from best to worst performing callers.

Figure 4A plot precision, sensitivity and F1 measures, when variants were accepted when called by a minimum intersection threshold (from 7 to 1) of 7 combined SNV callers. Figure 4B,C show the effect on F1 measure of increasing the number of combined variant callers, when callers are added in either ascending or descending order by individual caller performance.

### Amplicon sequencing

The noise returned by any single caller will not entirely overlap with a different caller, therefore noise should be reduced by accepting the intersections of combined callers. Counts of the remaining false positives are given in Table 2, when accepting calls made by a minimum number of callers. False positive counts were also totaled after removing the noisiest caller. However, this did not dramatically increase noise at minimum intersection thresholds n, n − 1, or n − 2.

**Table 2:** Counts of false positives called from 10 germline samples by a combination of 5 or 4 callers, across a range of minimum intersection thresholds.

	GL1	GL2	GL3	GL4	GL5	GL6	GL7	GL8	GL9	GL10
n = 5 callers Minimum intersection										
n	0	0	0	0	0	0	0	0	0	1
n − 1	0	0	0	1	1	1	1	1	1	3
n − 2	1	4	5	2	2	3	4	4	5	10
n − 3	18	26	20	12	18	15	15	17	17	44
n − 4	167	119	109	128	79	112	84	95	95	161
n = 4 callers Minimum intersection										
n	0	0	0	0	0	0	0	0	0	1
n − 1	0	0	1	1	1	1	1	1	1	3
n − 2	2	4	5	2	2	3	4	4	5	10
n − 3	100	102	97	70	72	95	78	92	63	101
Additional counts by 4 callers, at minimum intersection										
n	0	0	0	0	0	0	0	0	0	0
n − 1	0	0	1	0	0	0	0	0	0	0
n − 2	1	0	0	0	0	0	0	0	0	0

## Conclusions

In summary, the simple heuristics developed during this study for SNV calling are:

1. Multiple variant caller combination increases accuracy.
2. Accept variants called by n − 1 callers, where n is the total number of callers. That is, sensitivity is maintained by keeping the positives that are only (false) negative in a single caller.
3. Without prior knowledge of which variant callers are best suited to a dataset, accuracy may be increased by increasing the number of combined callers.
4. Removal of the worst performing caller from a combination does not necessarily increase accuracy

In contrast, indel calling requires more judicious selection and combination of variant callers:

1. Combination of two indel callers increases accuracy above that of either caller alone.
2. Accept variants called by both callers.
3. Addition of a third or fourth variant caller does not necessarily increase accuracy.

## Methods

### Whole genome sequencing

We aimed to combine a reasonably low number of variant callers while still covering a broad range of conceptual calling approaches by selecting callers representative of the 6 types of core variant callers algorithms identified in Xu's 2018 review of somatic variant callers^21^. The ‘machine learning’ core type was excluded when initial exploration suggested that variants called were overly dependent on data used for training, also termed ‘batch effects’^22^. A widely-used indel caller, Pindel^4^, was added as it takes a pattern growth approach unlike any of the 6 core algorithm types described by Xu. To prevent over-fitting and to enable replication, default parameters were used and parameter fine-tuning was avoided.

In their 2016 paper “A somatic reference standard for cancer genome sequencing”^20^, Craig et al*.* reported high coverage (99x) whole genome sequencing of a matched metastatic melanoma cell line (COLO829) and normal by Illumina HiSeq. We aligned the FASTQ files sequenced by Illumina to human genome version hg19/GRCh37 using BWA-MEM^23^, and called variants using the seven variant callers listed in Table 1.

Variants from the resultant VCF files were retained if their ‘FILTER’ field entry was either “PASS” or “.” and variant allele depth was > 3, and variant allele frequency > 0.02. This low stringency filtering was intended to remove some random “stochastic noise” without also removing the “deterministic noise" (noise/false positives systematically introduced by variant callers). The threshold was chosen after examination of a density histogram plotting variant allele depth of the caller that called the most variants, Pindel. The variants called by each caller were compared to the indels or SNVs of the Craig et al. reference standard using VCFtools^24^ and RTG tools^25^. Counts of true positives (TP), false positives (FP) and false negatives (FN) were used to calculate Precision TP/(TP + FP) and Sensitivity TP/(TP + FN) and their harmonic mean, the F1 measure. This study emphasizes maximizing F1, however maximizing sensitivity and minimizing false negatives may be preferred in clinical contexts such as biopsies of low tumor content, or investigation of specific implicated genes. We did not test for caller accuracy on low or very low frequency variants. All possible combinations of variant callers were tested, and variants were accepted if called by, or by more than, a minimum intersection of callers. That is, a minimum caller intersection threshold was applied to only accept calls made by at least a minimum of the number of callers. If n is the total number of combined callers, then this minimum intersection ranged between 2 to n. Every intersection threshold was tested in every possible combination of callers.

### Whole exome sequencing

The COLO829 somatic reference standard was produced from a cancer cell line, and lacks the complexity and heterogeneity of a bulk tumor sample. Therefore, we manually annotated^26^ read alignments pileups from the WES of a matched triple negative breast cancer sample (Supplementary Table 1). A sample of random locations were annotated as either containing a somatic, germline or no variant, or being ambiguous. 19 locations contained a somatic variant, and 1145 locations contained no variant. Because of this class imbalance, we generated another manually annotated reference by first filtering for locations with > 20 reads, > 5 variant reads, < 25% variant allele frequency in the matched germline normal sample and > 20% variant allele frequency called by at least two variant callers. A sample of these locations were manually annotated with 108 locations being labelled as containing a somatic variant, and 142 locations containing no variant which were treated as positives and negatives respectively in this WES reference set. Strelka^27^, a variant caller excluded from our previous WGS analysis due to its involvement in the pipelines used to create the WGS reference standard, was now added to the pool of variant callers. Somatic variant calls made by each caller are available in Supplementary Information as VCF files. Precision, sensitivity and F1 measures were calculated when variants were accepted as positive when called by more than a minimum number of the 7 SNV callers, and are plotted in Fig. 4.

### Amplicon sequencing

While the higher coverage of targeted amplicon sequencing can uncover lower frequency variants, PCR error increases background noise. PCR errors appearing in sequencing reads can look similar to real variants, making it difficult to manually annotate amplicon sequencing. Therefore, our study of variant caller combination in amplicon sequencing was focused on selectivity, and reduction of noise. We decided to utilize only somatic variant callers capable of calling both SNVs and indels. The five selected callers were: Mutect2, NextGene, Strelka2, VarDict and VarScan2. NextGene (SoftGenetics) is a software that includes a variant caller function tuned to the ThunderBolts Cancer Panel.

We evaluated the level of background noise by applying amplicon sequencing, using the ThunderBolts Cancer Panel (Bio-Rad), to ten germline normal samples. True germline allele frequencies can only be 0.5 (heterozygous) or 1 (homozygous). Noise variants called by each caller with frequency outside of 0.4–0.6 (heterozygous) or 0.9–1 (homozygous) were intersected. The noise returned by any single caller will not entirely overlap with a different caller, therefore by combining callers and accepting their intersections, a large amount of noise will be discarded. Counts of the remaining false positives are given in Table 2, when accepting calls made by a minimum number of callers. Somatic calls, as made in the previous WGS and WES analyses, had germline calls and shared noise subtracted, while no calls were subtracted from these germline calls.

## Supplementary Information

Supplementary Information 1.

Supplementary Information 2.

Supplementary Information 3.

## Data Availability

The WGS somatic reference standard of Craig et al*.* analysed during the current study are available in the National Center for Biotechnology Information database of Genotypes and Phenotypes (NCBI dbGAP, accession number phs000932, https://www.ncbi.nlm.nih.gov/projects/gap/cgi-bin/study.cgi?study_id=phs000932.v1.p1) and the European Bioinformatics Institute European Genome-phenome Archive (EBI EGA, accession number EGAS00001001385, https://ega-archive.org/studies/EGAS00001001385). The WES and amplicon sequencing datasets generated and analysed during the current study are not publicly available due to research participant privacy but are available from the corresponding author on reasonable request.
